# Barriers to Disaster Preparedness among Medical Special Needs Populations

**DOI:** 10.3389/fpubh.2015.00205

**Published:** 2015-09-02

**Authors:** Leslie Meyer, Kristina Vatcheva, Stephanie Castellanos, Belinda Reininger

**Affiliations:** ^1^University of Texas Health Science Center at Houston - School of Public Health, Brownsville Regional Campus, Brownsville, TX, USA

**Keywords:** Hispanics, disaster preparedness, evacuation barriers, medical special needs, hurricane

## Abstract

A medical special needs (MSN) assessment was conducted among 3088 respondents in a hurricane prone area. The sample was female (51.7%), Hispanic (92.9%), aged >45 years (51%), not insured for health (59.2%), and with an MSN (33.2%). Barriers to preparedness were characterized for all households, including those with inhabitants reporting MSN ranging from level 0 (mild) to level 4 (most severe). Multivariable logistic regression tested associations between hurricane preparedness and barriers to evacuation by level of MSN. A significant interaction effect between number of evacuation barriers and MSN was found. Among households that reported individuals with level 0 MSN, the odds of being unprepared increased 18% for each additional evacuation barrier [OR = 1.18, 95% CI (1.08, 1.30)]. Among households that reported individuals with level 1 MSN, the odds of being unprepared increased 29% for each additional evacuation barrier [OR = 1.29, 95% CI (1.11, 1.51)]. Among households that reported individuals with level 3 MSN, the odds of being unprepared increased 68% for each additional evacuation barrier [OR = 1.68, 95% CI (1.21, 1.32)]. MSN alone did not explain the probability of unpreparedness, but rather MSN in the presence of barriers helped explain unpreparedness.

## Introduction

Lack of preparedness for natural disasters stresses an already overwhelmed public health and medical system when disaster strikes (1, 2). For individuals, disaster preparedness involves the steps taken beforehand to identify and assemble adequate supplies for household members, including food, water, and medical needs, as well as establishing an emergency plan that should include possible evacuation shelters (3). However, lack of disaster preparedness is common and can increase potential damage, injury to self, and mortality (4). At the system level, there is a need for greater understanding of how organizations charged with managing disasters interact with the public before and during an emergency situation, and how these entities respond (1). For professionals responsible for directing public health disaster responses, preparedness includes identifying which populations may be most affected by the disaster and who will need priority responses to avert danger (5). Factors to consider include limited access and exposure to natural, technical, and social resources (5) and disproportionate access to medical care during extreme weather events (6).

The National Center for Emergency Medical (2) emphasizes that an effective and efficient evacuation requires the rapid assessment of individuals in need of assistance, the subsequent evacuation of those individuals, and their proper care (7). For example, individuals with limited financial resources may find it difficult to purchase extra food, water, or medications in preparation for a disaster and in the event of an evacuation. Others in need may include those with poor health, lack of social support networks, without knowledge of evacuation centers, without access to reliable transportation, or without knowledge of what to do in the event of an evacuation (8).

Almost 50%of adults in the US do not have emergency supplies in case of a disaster (9). This is particularly true among Hispanics who have been found to be the least prepared compared to other racial and ethnic groups (10–13). Previous studies have shown that Hispanics are less likely compared to Whites and Blacks to have a 3-day food supply, 3-day supply of medication, a working battery-operated radio, or a flashlight (11). Primarily Spanish-speaking Hispanics, compared to primarily English-speaking Hispanics also report lower levels of preparation in these four categories, but are more likely to have an emergency evacuation plan and 3-day supply of water (11). Differences in disaster preparedness have been documented (11–15), but few studies have examined if the presence of medical special needs (MSN) influences disaster preparedness.

The Special Needs Assessment for Katrina Evacuees (SNAKE) project, conducted by the National Organization on Disability, shed light on numerous barriers and policies associated with negative experiences shared by individuals with disabilities who evacuated to shelters when Hurricane Katrina struck the Gulf Coast in 2005 (16). Barriers included access to reliable and accessible shelters with protocols in place to provide services for individuals with disabilities and MSN (16). Individuals with MSN have decreased access to appropriate transportation for evacuation (17), may require additional equipment that would need transportation (18), and have limited access to an appropriate host shelter due to the level of care required (17).

Research on vulnerable populations and disaster preparedness geographically is limited (10, 19, 20). Previous studies have surveyed the general Texas population for evacuation intentions and experiences (21, 22). An assessment of disaster preparedness determined that slightly more than half (59%) of residents in this three county area known as the Texas Rio Grande Valley stated that they were prepared for a hurricane (13). Previous research has not reported differences between disaster preparedness and MSN levels of vulnerability among a population in a disaster prone region.

This study aims to characterize disaster preparedness and its barriers among vulnerable populations living in a hurricane prone area. Specifically, we will compare disaster preparedness and barriers to evacuation across households reporting MSN and households without MSN. We hypothesize that households with higher levels of MSN will experience more barriers to evacuation and less preparedness for disasters.

## Materials and Methods

### Sample

The study sample included household respondents surveyed as part of the Medical Special Needs Assessment of the Lower Rio Grande Valley (23). The survey was administered to 3088 individuals in 172 census tracts and was weighted to represent the 696,349 individuals across Cameron, Hidalgo, and Willacy Counties, Texas. These are counties located on or near the Texas Coastline, an area prone to hurricanes. The methods for data collection have previously been reported (12, 23).

The assessment was conducted by trained community health workers who interviewed, with signed informed consent, one adult per household. Thirty surveys were completed from each of 100 census tracts and were administered in English or Spanish, depending upon the respondent’s preferred language.

### Measurement of medical special needs

Previously defined levels of MSN, customary in the state of Texas in 2007 (23), are described in Table 1 and were measured in the current study. MSN level 5 includes individuals in institutional settings, such as hospitals, long-term care facilities, assisted living facilities, or state schools, and is not assessed in the present study because data collection was conducted solely in homes.

**Table 1:** **Level of medical special needs (MSN)**.

No special needs: no medical needs and no required assistance
Level 0: no medical needs, but require transportation assistance for evacuation
Level 1: dependent on others for routine care (eating, walking, toileting, etc.) and children under 18 without adult supervision
Level 2: physical or developmental disabilities, such as blindness, significant hearing impairment, amputation, deafness, and mental retardation
Level 3: require assistance with medical care administration, monitoring by nurse, dependent on equipment, assistance with medications, and mental health disorders
Level 4: persons outside an institutional facility care setting, who require extensive medical oversight (i.e., IV, chemotherapy, life support equipment, morbidly obese)

*Levels defined as by the State of Texas Hurricane Evacuation and Mass Care Plan, 2007*.

### Measurement of disaster preparedness

To assess disaster preparedness, respondents were asked “How prepared are you if a major hurricane were to strike your community during the next hurricane season?” Responses of “very” and “somewhat” were identified as “prepared” for a hurricane. Responses of “not too” and “not at all” were identified as “not prepared” for a hurricane.

### Measurement of barriers

A total of 15 barriers to evacuation for a hurricane were assessed based on a previous instrument (24). A scale score was calculated by summing the number of affirmative responses to each barrier. This scale score was used in multivariable analysis models.

### Sociodemographic variables

Sociodemographic variables used to characterize the population included gender, age, educational attainment (elementary/middle school, high school, or college/technical), marital status (married or not married), ethnicity, acculturation (high adherence with Spanish, biculturalism, or high adherence with English), health status (poor, fair, good, very good, or excellent), health insurance, homeowner/renter’s insurance, household size, household income, adults in household ages 65 years and older, children in household under the age of 10, cash savings, and distance to shore.

### Statistical analysis

Population estimates for the parameters and their SEs were generated by taking into account the study design features and incorporating the sampling weights. For descriptive purposes, categorical variables were summarized in unweighted frequencies and weighted percentages. Continuous variables were summarized using weighted means and their SEs. In univariable weighted logistic regression analysis, we determined association between variables and the dichotomized variable for self-reported preparedness. Results are presented with weighted odds ratios and their 95% confidence intervals. In addition, an ordinal variable with the six levels of MSN was created. Since the assumptions for ordinal logistic regression did not hold, weighted multinomial logit models were fitted to obtain the odds of higher levels of MSN versus no MSN. We built a weighted multivariable logistic regression model for self-reported preparedness (no/yes), including variables with Rao-Scott design-adjusted chi-square test statistics *p*-value <0.10 from the univariable analysis. Harmful multicollinearity between the independent variables considered in the model was not found (VIF < 1.5). The linearity assumption in the logit for continuous variables was checked. The contribution of interactions to the fit of the model was tested using design-adjusted Wald test significance level *p* < 0.05 and the statistically non-significant interactions were removed from the model. The preliminary final model was tested for goodness-of-fit applying Archer and Lemeshow’s *F*-adjusted mean residual goodness-of-fit test using (25). All other weighted analyses were conducted using (26). All statistical testing was two-sided and was performed using a significance (alpha) level of 0.05.

## Results

The final sample for this study consisted of a total of 2981 respondents (Table 2). Slightly more than half of respondents were female (51.7%), aged 45 years or older (51%), with a mean age of 46.9 (SE 0.79). Only 18.3% of respondents had a college or technical education and 73.5% were married. The sample was predominantly Hispanic (92.9%) with high acculturation levels of adherence to the Spanish language (58.4%). A low percentage (5.1%) of respondents was in excellent health, 59.2% were without any form of health insurance, and 61.4% were homeowner uninsured. The majority household size was between 3 and 5 people (58.7%) with a household income below $35,000 (87.6%). Among households, 12.9% had at least one adult aged 65 or older; 40.8% had at least one child under the age of 10; 40.8% had at least $300 in cash savings; the mean distance from the shore was 41.6 miles (SE 1.03); and 66.8% had no MSN household members.

**Table 2:** **Demographics characteristics of respondents and households and reported barriers to evacuation (*n* = 2981)**.

	*n* (weighted %)	Total number of evacuation barriers Mean (SE)	Difference in mean *p*-Value
**RESPONDENT LEVEL**			
**Gender**
Male	760 (48.3)	5.8 (0.23)	0.6728
Female^a^	2221 (51.7)	5.9 (0.12)	
**Age**
18–44 years^a^	1460 (49.0)	5.8 (0.23)	0.7899
≥45 years	1520 (51.0)	5.9 (0.12)	
**Education**
≤8 years	1123 (38.5)	6.2 (0.16)	0.0406
High school	1235 (43.2)	5.7 (0.22)	0.5145
College/technical^a^	529 (18.3)	5.4 (0.35)	
**Marital status**
Not married	901 (26.5)	5.5 (0.21)	0.003
Married^a^	2080 (73.5)	6.0 (0.16)	
**Ethnicity**
Hispanic	2738 (92.9)	5.9 (0.17)	0.1317
Non-Hispanic^a^	243 (7.1)	5.1 (0.52)	
**Acculturation**
Spanish	1780 (58.4)	6.0 (0.14)	0.315
Bicultural	903 (32.1)	5.8 (0.26)	0.5429
English^a^	267 (9.5)	5.4 (0.50)	
**Health condition**
Poor	65 (1.9)	6.4 (0.33)	0.0002
Fair	537 (15.4)	6.2 (0.26)	<0.0001
Good	1677 (57.4)	6.0 (0.13)	<0.0001
Very good	518 (20.1)	5.6 (0.38)	0.0071
Excellent^a^	163 (5.1)	4.4 (0.39)	
**Health insurance**
Insured	1213 (40.8)	5.7 (0.21)	0.3388
Not insured^a^	1758 (59.2)	5.9 (0.18)	
**Homeowner’s insurance**
Insured	1140 (38.6)	5.6 (0.23)	0.1927
Uninsured^a^	1620 (61.4)	6.0 (0.21)	
**HOUSEHOLD LEVEL**			
**Household size**
1–2 people	710 (24.0)	5.3 (0.21)	<0.0001
3–5 people	1737 (58.7)	5.9 (0.20)	0.0021
>5 people^a^	527 (17.4)	6.5 (0.17)	
**Income**
<10,000	902 (32.8)	6.2 (0.18)	0.1262
10,000 to <15,000	676 (27.9)	6.1 (0.30)	0.1944
15,000 to <25,000	394 (17.5)	5.7 (0.22)	0.421
25,000 to <35,000	217 (9.7)	5.7 (0.32)	0.4481
≥35,000^a^	278 (12.4)	5.3 (0.53)	
**Adults >65 years**
≥1 person	430 (12.9)	6.0 (0.19)	0.3029
**Children <10 years**
≥1 person	1270 (41.6)	5.9 (0.18)	0.2976
**Cash savings**
≥$300	1164 (40.8)	5.6 (0.21)	0.0527
**Level of medical needs**
No medical needs	1976 (66.8)	5.5 (0.19)	0.1023
Level 0	500 (16.1)	6.4 (0.23)	0.9401
Level 1	251 (9.2)	6.0 (0.34)	0.4623
Level 2	66 (2.0)	5.9 (0.38)	0.4195
Level 3	83 (3.3)	8.4 (0.46)	0.0008
Level 4^a^	78 (2.6)	6.4 (0.53)	

*^a^Reference group*.

On average respondents with <8 years of education had significantly higher numbers of evacuation barriers (mean 6.2, SE 0.16, *p* = 0.0406) in comparison to those with a college or technical education (mean 5.4, SE 0.35) (Table 2). In addition, respondents at any level of self-reported health conditions (poor: *p* = 0.0002, fair: *p* < 0.0001, good: *p* < 0.0001, very good: *p* = 0.0071) reported significantly higher average number of evacuation barriers compared to those with excellent self-reported health. Smaller households of size 1–2 people (*p* < 0.0001) or 3–5 people (*p* = 0.0021) reported significantly lower average number of evacuation barriers compared to households with more than 5 people. Households reporting an individual requiring assistance with medical care administration (MSN Level 4) reported significantly higher average number of evacuation barriers (mean 8.4, SE 0.46) compared to households reporting an individual requiring extensive medical oversight (MSN Level 5) (mean 6.4, SE 0.53; *p* = 0.0008).

Using univariable logistic regression analyses, the odds of being unprepared for a hurricane (Table 3) were 1.63 times higher for respondents aged between 18 and 44 years [95% CI (1.30, 2.05)] than the odds for respondents aged 45 years or older. Similarly, the odds of being unprepared were higher for those with 8 years of education or less [OR = 1.45, 95% CI (1.01, 2.10)] or a high school diploma [OR = 1.48, 95% CI (1.04, 2.10)] compared to those with a college or technical education. In addition, respondents who reported having health insurance were 33% [OR = 0.67, 95% CI (0.51, 0.88)] more prepared compared to those without health insurance, and respondents with homeowner’s insurance were 40% [OR = 0.60, 95% CI (0.45, 0.80)] more prepared compared to those without homeowner’s insurance.

**Table 3:** **Individual level characteristics by hurricane preparedness**.

	*n* (weighted %)	Not prepared (weighted %)	Prepared (weighted %)	OR (95% CI)
**Gender**
Male	747 (40.5)	268 (59.5)	479 (50.2)	0.85 (0.70, 1.03)
Female^a^	2176 (51.5)	907 (45.58)	1269 (55.5)	
**Age**
18–44	1442 (49.3)	652 (48.6)	790 (51.4)	**1.63 (1.30, 2.05)**
45–75+^a^	1480 (50.7)	522 (36.7)	958 (63.3)	
Mean (SE)	46.9 (0.8)	44.0 (1.0)	49.0 (0.8)	**0.98 (0.975, 0.989)**
**Education**
≤8 years	1097 (38.5)	459 (44.2)	638 (55.8)	**1.45 (1.01, 2.09)**
HS diploma	1213 (43.2)	508 (44.6)	705 (55.4)	**1.48 (1.04, 2.10)**
College/technical^a^	521 (18.3)	173 (35.3)	348 (64.7)	
**Marital status**
Not married	877 (26.3)	353 (40.7)	524 (59.3)	0.90 (0.72, 1.12)
Married^a^	2046 (73.7)	882 (43.3)	1224 (56.7)	
**Ethnicity**
Hispanic	2681 (92.8)	1101 (43.5)	1580 (56.5)	1.68 (0.99, 2.86)
White^a^	242 (7.2)	74 (31.3)	168 (68.7)	
**Acculturation**
Spanish	1748 (58.6)	750 (45.6)	998 (54.4)	1.32 (0.81, 2.14)
Bicultural	880 (31.8)	332 (38.1)	548 (61.9)	0.97 (0.59, 1.58)
English^a^	266 (9.6)	82 (38.9)	184 (61.1)	
**Health condition**				
Poor	58 (1.8)	31 (50.0)	27 (50.0)	1.90 (0.84, 4.30)
Fair	526 (15.3)	233 (47.7)	293 (52.3)	1.73 (0.98, 3.06)
Good	1641 (57.3)	668 (43.6)	973 (56.4)	1.47 (0.86, 2.51)
Very good	515 (20.4)	185 (36.8)	330 (63.2)	1.11 (0.65, 1.89)
Excellent^a^	162 (5.2)	48 (34.5)	114 (65.5)	
**Health insurance**				
Insured	1184 (40.6)	411 (36.9)	773 (63.1)	**0.67 (0.51, 0.88)**
Uninsured^a^	1729 (59.4)	762 (46.6)	967 (53.4)	
**Homeowner’s insurance**				
Insured	1114 (38.4)	357 (34.6)	757 (65.4)	**0.60 (0.45, 0.80)**
Uninsured^a^	1597 (61.6)	717 (47.0)	880 (53.0)	

*Bold font indicates statistical significance*.

*SE, standard error; HS, high school; HA, high adherence*.

*^a^Reference group*.

*The percentages, odds ratios, and 95% confidence intervals are weighted*.

Household size also had a significant effect on preparedness (Table 4). Respondents of smaller households, 1–2 people, or 3–5 people were 40% [95% CI (0.28, 0.56)] and 58% [95% CI (0.46, 0.73)], respectively, less likely to be unprepared compared to larger households of more than 5 people. Households with lower annual income were more likely to be unprepared compared to households with an income >$35,000. Respondents reporting higher numbers of evacuation barriers were significantly more likely to be unprepared for a hurricane. With a 1 count increase in number of evacuation barriers, the expected increase in the odds of being unprepared was 3%. The evacuation barriers of “the entire family cannot leave,” “think the roads would be too crowded to leave,” “worry possessions would be stolen or damaged,” “cannot afford to leave (travel expenses),” “think evacuating will be dangerous,” “will be safe at home,” and “unable to work will mean being replaced” were significantly associated with evacuation unpreparedness, where very high odds of being unprepared were observed in respondents where “the entire family cannot leave” [OR = 2.62, 95% CI (2.00, 3.40)], in respondents who “think the roads would be too crowded to leave” [OR = 2.37, 95% CI (1.76, 3.19)], and in respondents who reported that being “unable to work will mean being replaced” [OR = 2.25, 95% CI (1.55, 3.27)]. Additionally, respondents reporting a higher level of MSN were more likely to be unprepared compared to those reporting no MSN. Specifically, the odds of respondents reporting an individual requiring assistance with medical care administration (MSN Level 4) were 3.82 times higher [95% CI (2.08, 7.02)] to be unprepared for an evacuation compared to those reporting no MSN. Likewise, respondents reporting an individual who depends on others for routine care (MSN Level 1) were 2.46 times more likely to be unprepared [95% CI (1.64, 3.70)] compared to those with no MSN. Respondents reporting an individual requiring transportation assistance (MSN Level 0) were 1.88 times more likely to be unprepared [95% CI (1.37, 2.58)] compared to those with no MSN.

**Table 4:** **Household level characteristics and barriers by hurricane preparedness**.

	*n* (weighted %)	Not prepared (weighted %)	Prepared (weighted %)	OR (5% CI)
**Household size**
1–2 people	688 (23.6)	233 (33.4)	455 (66.6)	**0.40 (0.28, 0.56)**
3–5 people	1710 (59.0)	680 (42.3)	1030 (57.7)	**0.58 (0.46, 0.73)**
>5 people^a^	519 (17.4)	260 (56.0)	259 (44.0)	
**Income**
<$10,000	878 (32.3)	378 (45.4)	500 (54.6)	**2.13 (1.32, 3.42)**
$10,000–$14,999	661 (27.9)	301 (48.5)	360 (51.5)	**2.41 (1.50, 3.89)**
$15,000–$24,999	394 (17.8)	145 (40.6)	249 (59.4)	**1.75 (1.11, 2.76)**
$25,000–$34,999	216 (9.7)	83 (45.0)	133 (55.0)	**2.09 (1.22, 3.57)**
≥$35,000^a^	274 (12.4)	77 (28.1)	197 (71.9)	
**Adults >65 years**
≥1 person	414 (12.8)	127 (32.0)	287 (68.0)	**0.59 (0.44, 0.80)**
**Children <10 years**
≥1 person	1256 (41.9)	564 (48.0)	692 (52.0)	**1.46 (1.15, 1.85)**
**Cash savings**
≥$300	1145 (41.0)	325 (32.7)	820 (67.3)	**0.50 (0.39, 0.63)**
**Distance from shore (miles)**
Mean (SE)	41.6 (1.02)	46.6 (1.1)	38.4 (1.1)	**1.03 (1.03, 1.04)**
**Evacuation barriers**
1. The entire family cannot leave	2016 (72.6)	943 (48.6)	1073 (51.4)	**2.62 (2.00, 3.40)**
2. Think road would be too crowded to leave	1991 (71.1)	909 (48.4)	1082 (51.6)	**2.37 (1.76, 3.19)**
3. Worry possessions stolen/damaged	1971 (69.5)	836 (44.5)	1135 (55.5)	**1.31 (1.04, 1.65)**
4. Cannot afford to leave (travel expenses)	1620 (56.9)	710 (45.4)	910 (54.6)	**1.30 (1.01, 1.68)**
5. Do not know where to go	1564 (55.5)	662 (45.0)	902 (55.0)	1.25 (0.96, 1.62)
6. Do not have transportation	1287 (46.7)	542 (43.8)	745 (56.2)	1.10 (0.83, 1.45)
7. Think shelters might be unsafe or unsanitary	1227 (40.5)	492 (42.1)	735 (57.9)	0.97 (0.77, 1.21)
8. Have medical/physical problems	432 (12.8)	177 (46.0)	255 (54.0)	1.18 (0.88, 1.56)
9. Think evacuating will be dangerous	1146 (41.8)	530 (50.2)	616 (49.8)	**1.71 (1.30, 2.25)**
10. Work during hurricane	391 (12.7)	135 (36.3)	256 (63.7)	0.74 (0.51, 1.07)
11. Do not want to leave pet	893 (31.0)	367 (45.0)	526 (55.0)	1.15 (0.83, 1.62)
12. Will be safe at home	1269 (45.5)	584 (49.1)	685 (50.9)	**1.63 (1.21, 2.21)**
13. Unable to work will mean being replaced	453 (16.4)	231 (59.3)	222 (40.7)	**2.25 (1.55, 3.27)**
14. No proper documents to leave area	1213 (45.8)	500 (42.5)	713 (57.5)	1.00 (0.76, 1.31)
15. Care for someone who cannot leave	320 (9.5)	118 (42.0)	202 (58.0)	0.98 (0.69, 1.38)
**No. evacuation barriers**
Mean (SE)	5.8 (0.2)	6.4 (0.2)	5.4 (0.2)	**1.13 (1.07, 1.19)**
**Level of medical needs**
No medical needs^a^	1932 (66.7)	681 (36.6)	1251 (63.4)	**1.88 (1.37, 2.58)**
Level 0	498 (16.3)	241 (52.0)	257 (48.0)	**2.46 (1.64, 3.70)**
Level 1	245 (9.1)	137 (58.7)	108 (41.3)	1.52 (0.78, 2.96)
Level 2	65 (2.0)	25 (46.7)	40 (53.3)	**3.82 (2.08, 7.02)**
Level 3	82 (3.3)	50 (68.8)	32 (31.2)	1.19 (0.65, 2.19)
Level 4	75 (2.6)	28 (40.8)	47 (59.2)	

*Bold font indicates statistical significance*.

*SE, standard error*.

*^a^Reference group*.

*The percentages, odds ratios, and 96% confidence intervals are weighted*.

Univariable multinomial logistic regression analyses tested the individual, household, and evacuation barriers by MSN (data not shown). The odds of level 0 MSN versus no MSN was 26% lower for males compared to females [OR = 0.74, 95% CI (0.55, 1)]. We also found the odds of level 0 MSN versus no MSN toss be 71% lower in respondents aged greater than 45 years in comparison to those aged 44 years or younger [OR = 0.29, 95% CI (0.21, 0.41)]. The odds of level 0 MSN versus no MSN was 35% lower for unmarried respondents compared to married respondents [OR = 0.65, 95% CI (0.48, 0.9)]. Respondents reporting higher numbers of evacuation barriers were significantly more likely to be level 0 MSN. With a 1 number increase in evacuation barriers, an expected increase in the odds of reporting level 0 MSN was 13% [OR = 1.13, 95% CI (1.05, 1.21)]. Higher odds of reporting level 0 MSN were observed when respondents also reported “the entire family cannot leave” [OR = 6.58, 95% CI (3.73, 11.58)], or that “the roads would be too crowded to leave” [OR = 2.63, 95% CI (1.75, 3.96)], or “unable to work will mean being replaced” [OR = 3.03, 95% CI (2.00, 4.59)].

The multivariable logistic regression model for the probability of being unprepared for a hurricane (Table 5) shows that after adjusting for ethnicity, adults in household 65 years or older, cash savings of at least $300, income, age of respondent by household size, distance from shore and level of MSN and number of evacuation barriers, the odds of Hispanics to be unprepared for a hurricane were 49% lower in comparison to non-Hispanics [OR = 0.51, 95% CI (0.27, 0.95)]. In addition households with incomes between $25,000 and $35,000 had 1.96 [95% CI (1.13, 3.39)] times higher odds of being unprepared compared to those with income greater than $35,000. The odds of households with at least one adult aged 65 years or older to be unprepared is 32% [OR = 0.68, 95% CI (0.47, 0.98)] lower compared to households with no adults aged 65 years or older. Additionally, there was a significant statistical interaction between age and household size, where the effect of age on unpreparedness was significant only in household sizes with 1–2 people [OR = 0.98, 95% CI (0.97, 0.99)]. This result indicates that for every year increase in age of respondents among households with 1–2 people, there were slightly decreased odds of being unprepared. For every one mile increase in distance from the shore, the odds of being unprepared increased by 3% [OR = 1.03, 95% CI (1.02, 1.04)].

**Table 5:** **Multivariable logistic regression model for probability of being unprepared for effects**.

	Unprepared for hurricane OR (95% CI)
**RESPONDENT LEVEL**	
**Ethnicity**
Hispanics	**0.51 (0.27, 0.95)**
Non-Hispanics^a^	
**Adults >65 years**
≥1 person	**0.68 (0.47, 0.98)**
No adults >65^a^	
**Cash savings**
≥$300	**0.60 (0.48, 0.74)**
<$300^a^	
**Income**
<$10,000	1.52 (0.97, 2.37)
$10,000–$14,999	1.39 (0.87, 2.22)
$15,000–$24,999	1.16 (0.74, 1.83)
$25,000–$34,999	**1.96 (1.13, 3.39)**
≥$35,000^a^	
**HOUSEHOLD LEVEL**	
**Age of respondent by household size**	
Age of respondent in household of 1–2 people	**0.98 (0.97, 0.99)**
Age of respondent in household of 3–5 people	1.01 (0.99, 1.03)
Age of respondent in household of >5 people	1 (0.99, 1.01)
**Distance from shore (miles)**	**1.03 (1.02, 1.04)**
**Level of medical special needs**	
Number of evacuation barriers in individuals with No MSN	0.98 (0.93, 1.03)
Number of evacuation barriers in individuals with Level 0 MSN	**1.18 (1.08, 1.30)**
Number of evacuation barriers in individuals with Level 1 MSN	**1.29 (1.11, 1.51**)
Number of evacuation barriers in individuals with Level 2 MSN	1.14 (0.86, 1.52)
Number of evacuation barriers in individuals with Level 3 MSN	**1.68 (1.21, 2.32)**
Number of evacuation barriers in individuals with Level 4 MSN	0.96 (0.79, 1.16)

*Bold font indicates statistical significance*.

*MSN, medical special needs*.

*^a^Reference group*.

A significant interaction between the number of evacuation barriers and the levels of MSN resulted in varying effects of being unprepared across the MSN levels. Among households with individuals requiring transportation assistance (MSN Level 0), for every one additional increase in the number of evacuation barriers the odds of being unprepared increased by 18% [OR = 1.18, 95% CI (1.08, 1.30)]. Among households that reported individuals who depend on others for routine care (MSN Level 1), for every one additional increase in the number of evacuation barriers the odds of being unprepared increased by 29% [OR = 1.29, 95% CI (1.11, 1.51)]. Also, among households that reported individuals who require assistance with medical care administration (MSN Level 3), for every one additional increase in the number of evacuation barriers the odds of being unprepared increased 68% [OR = 1.68, 95% CI (1.21, 1.32)].

## Discussion

This study examined preparedness for a hurricane and barriers to evacuation among households reporting MSN and households without MSN along the South Texas Gulf Coast. We hypothesized that households with higher levels of MSN would experience more barriers to evacuation and less preparedness for disasters. Our hypothesis was not proven because the effect of MSN on preparedness is more complex than our proposed simplistic linear hypothesis.

Medical special needs alone did not explain the probability of unpreparedness, but rather MSN in the presence of barriers played a role in explaining unpreparedness. As the number of barriers increased, households reporting the presence of individuals with MSN of levels 0, 1, and 3 were significantly more likely to be unprepared, but this interaction effect was not significant in households reporting the presence of individuals with levels 2 and 4 MSN and among households with no MSN members. Therefore, it was not as simple as the higher the level of MSN the greater the unpreparedness. Likewise, there is no simple explanation for these findings, but rather we propose potential scenarios to shed light on the complexities. For households (MSN Level 0) that need transportation assistance during an evacuation, the barriers of feeling the family will be safe at home and not having travel expenses among other barriers may have led respondents to report being unprepared for a hurricane. For households (MSN Level 1) with either small children or adults who need help with routine care, the barrier of the roads will be too crowded to leave may be tied to respondents reporting being unprepared for a hurricane. Finally, for households (MSN Level 3) that have individuals who are monitored by a nurse, receive medication assistance, are dependent on equipment, or have a mental health disorder, the barrier of the entire family not being able to leave, in addition to other barriers, may have influenced respondents reporting being unprepared for a hurricane. In essence, it appears that once a household reports MSN where individuals are primarily dependent on others in the household for care or have medication or equipment needs and the barriers increase, the result is a greater likelihood of unpreparedness. However, we did not find that level 2 (physical or developmental disabilities) or level 4 (extensive medical oversight) reported the same level of unpreparedness in the face of barriers. It is possible that households with individuals at level 2 and level 4 are more likely to receive aid from government institutions and local entities. For example, individuals with MSN level 2 (mental retardation, amputations, and blindness) have access to some services provided through the State Department of Health and Human Services. For individuals with level 4 MSN (requiring extensive medical oversight in the home), some level of access to services has already been obtained by these households and it is possible that preparedness plans are part of that medical oversight care. Further exploration of the interactions between barriers and MSN is needed not only in this Gulf Coast region where preparedness for hurricanes is essential but also in other geographic regions of the country and world where individuals with MSN may be at greater risk for morbidity or mortality in the face of a disaster.

We found that Hispanics were generally less likely to be unprepared for a hurricane than the non-Hispanic population in the region. Over 50% of the sample preferred Spanish language. Past research has found that primary Spanish-speaking Hispanics are more likely to report having an emergency evacuation plan although less likely to have the supplies necessary to survive during a disaster (11). Households with at least one person aged 65 years or older, and with less cash on hand were less likely to be unprepared for a hurricane. These results may indicate that individuals in those particular households have developed the networks and support needed to manage and create a sense of preparedness for hurricanes. We also found that as respondents’ age increases in smaller households, the respondents indicated less unpreparedness. It is also possible that households with older individuals have faced and survived past hurricanes and therefore have a more accurate depiction of their preparedness for a hurricane. Finally, our results indicate that respondents living in households further from the shoreline indicated greater levels of unpreparedness. It is likely that this is a function of the belief that inland locations are safer from hurricane devastation than shoreline locations, and while not always an accurate belief could be an explanation of this finding.

Our study has limitations. One is that the surveys were based on interview and self-reported data and therefore potentially introduced respondent bias. The cross-sectional study design also limits any causation inferences. This study is based on a specific geographical location with the sample comprised predominately of low income Hispanic households and thus may not be generalizable to other geographic regions or populations. Also, respondents answered evacuation barriers in response to hurricanes which are prominent in the area and may not be representative of barriers to a different natural disaster or emergency evacuation situation.

Future research should examine the role support systems have in preparedness for individuals with MSN. Not all individuals who need assistance or qualify for assisted living are housed in a facility. While we found that 33.2% of respondents in our study sample reported at least one individual with MSN (Level 0–4) in the household, we expect that this is a higher rate of MSN found in home settings because of high levels of poverty and lack of health insurance found in this region. Future research also should characterize the strategies and effectiveness of the public health system’s response to the evacuation needs of the MSN population. This characterization could identify gold standard evacuation processes for MSN and foster their dissemination broadly.

## Conclusion

Certain MSN populations in light of increasing barriers reported unpreparedness for hurricanes in a region of the country prone to such natural disasters. Our results characterize a population vulnerable to hurricanes, and highlight variables and interactions among variables influential in preparedness. This study contributes new understanding of preparedness among MSN populations.

## Authors Contributions

LM, SC, and BR conceptualized the research hypothesis. KV conducted statistical analysis and LM, KV, SC, and BR interpreted the data. LM, KV, SC, and BR drafted the manuscript. All authors read and approved the final manuscript.

## Conflict of Interest Statement

The authors of this manuscript declare no conflict of interest. There have been no payments or services for the work of this manuscript. There is no financial relationships with any entities that may have influenced the work of this manuscript. There are no declarations for patent or copyright for work related to this manuscript.
